# Patient preferences for investigating cancer-related symptoms in Australian general practice: a discrete-choice experiment

**DOI:** 10.3399/BJGP.2023.0583

**Published:** 2024-07-16

**Authors:** Brent Venning, Alison Pearce, Richard De Abreu Lourenco, Rebekah Hall, Rebecca J Bergin, Alex Lee, Keith Donohoe, Jon Emery

**Affiliations:** Department of General Practice and Primary Care, Melbourne Medical School, University of Melbourne, Melbourne, Australia, and Centre for Cancer Research, University of Melbourne, Melbourne, Australia.; Daffodil Centre, University of Sydney, a joint venture with Cancer Council NSW, Sydney, Australia, and Sydney School of Public Health, University of Sydney, Sydney, Australia.; Centre for Health Economics Research and Evaluation, University of Technology Sydney, Haymarket, Australia.; Acaster Lloyd Consulting Ltd, London, UK.; Department of General Practice and Primary Care, Melbourne Medical School, University of Melbourne, Melbourne, Australia; Centre for Cancer Research, University of Melbourne, Melbourne, Australia; and Cancer Epidemiology Division, Cancer Council Victoria, Melbourne, Australia.; Department of General Practice and Primary Care, Melbourne Medical School, University of Melbourne, Melbourne, Australia, and Centre for Cancer Research, University of Melbourne, Melbourne, Australia.; Consumer Advisory Committee, Victorian Comprehensive Cancer Centre, Melbourne, Australia.; Department of General Practice and Primary Care, Melbourne Medical School, University of Melbourne, Melbourne, Australia, and Centre for Cancer Research, University of Melbourne, Melbourne, Australia.

**Keywords:** cancer, diagnostic tests, general practice, investigations, patient preference, symptoms

## Abstract

**Background:**

Striking the right balance between early cancer diagnosis and the risk of excessive testing for low-risk symptoms is of paramount importance. Patient-centred care must also consider patient preferences for testing.

**Aim:**

To investigate the diagnostic testing preferences of the Australian public for symptoms associated with oesophagogastric (OG), bowel, or lung cancer.

**Design and setting:**

One of three discrete-choice experiments (DCEs) related to either OG, bowel, or lung cancer were administered to a nationally representative sample of Australians aged ≥40 years.

**Method:**

Each DCE comprised three scenarios with symptom positive predictive values (PPVs) for undiagnosed cancer ranging from 1% to 3%. The numerical risk was concealed from participants. DCE attributes encompassed the testing strategy, GP familiarity, test and result waiting times, travel duration, and test cost. Preferences were estimated using conditional and mixed logit models.

**Results:**

A total of 3013 individuals participated in one of three DCEs: OG (*n* = 1004), bowel (*n* = 1006), and lung (*n* = 1003). Preferences were chiefly driven by waiting time and test cost, followed by the test type. There was a preference for more invasive tests. When confronted with symptoms carrying an extremely low risk (symptom PPV of ≤1%), participants were more inclined to abstain from testing.

**Conclusion:**

Access-related factors, particularly waiting times and testing costs, emerged as the most pivotal elements influencing preferences, underscoring the substantial impact of these systemic factors on patient choices regarding investigations.

## Introduction

In everyday clinical practice, GPs encounter a wide array of patient presentations and must efficiently determine, within a limited timeframe, who requires additional testing, which specific tests are most appropriate, and who can be monitored without immediate investigation. In Australia, GPs have the authority to directly refer patients for various pathology and radiology tests, as well as specific diagnostic procedures such as gastrointestinal endoscopy, which are accessible through open-access programmes.

Direct access to diagnostic tests from Australian primary care has been associated with earlier cancer diagnoses and improved cancer survival rates.^1^ However, this occurs within a complex system that encompasses elements of both public and private health care. Public hospital services are free at the point of care, whereas, for services outside of hospitals, Medicare, the national health insurance scheme, offers fixed benefits.^2^ Patients may incur additional charges, known as ‘out-of-pocket costs’, when healthcare providers charge fees exceeding the fixed benefits.^2^ In 2019, around 20% of individuals undergoing diagnostic imaging faced out-of-pocket expenses.^3^ In addition to this, patients with private health insurance have the option to receive care within private hospitals, giving them the choice to bypass waiting lists in the public system. Approximately 45% of Australians have private health insurance; nearly two out of every five admissions to hospital and around 75% of colonoscopies are conducted in the private sector.^4^^–^^6^

Patient preferences for diagnostic testing within this broader system context have not been well explored. There is ample literature on patient preferences for cancer screening tests; however, research specifically focused on preferences for symptomatic investigations is limited.^7^^,^^8^ Previous vignette studies have found that patients want their symptoms investigated at low levels of cancer risk regardless of cancer type, treatment options, or prognosis.^9^^,^^10^ However, these studies informed participants that low-risk symptoms may specifically indicate cancer, presented numerical risk estimates, offered only a single testing option or no test, and did not account for the impact of service delivery factors on choice.^9^^,^^10^ One qualitative study on preferences for lower gastrointestinal (GI) investigations found a higher priority was placed on the quality and accuracy of tests compared with test invasiveness despite a tendency for patients to underestimate the risks associated with investigations and overestimating their potential benefits.^11^^,^^12^ Importantly, focusing solely on clinical factors ignores the broader range of structural (for example, waiting times, travel distances), process (for example, effective communication, continuity of care), attitudinal, and sociodemographic factors that can significantly impact on how patients formulate preferences for different diagnostic strategies.^8^^,^^13^

**Table:** How this fits in

Achieving a delicate balance between early cancer diagnosis and avoiding excessive testing for common low-risk symptoms is crucial for patient-centred care. Although ample literature addresses patient preferences for cancer screening, limited research focuses on preferences for symptomatic investigations. The current study emphasises that preferences are primarily influenced by waiting time and test cost followed by the invasiveness of the test. Notably, participants lean towards abstaining from testing when faced with symptoms carrying an extremely low risk.

Discrete-choice experiments (DCEs) are a method used to quantitatively estimate preferences. DCEs present participants with a decision-making scenario followed by a series of choice tasks where they are asked to choose between two or more options defined by their characteristics, termed attributes. DCEs assume that individuals choose the option that provides them with the greatest value or ‘utility’.^14^ Each participant completes multiple-choices tasks and, by analysing the patterns of these choices, researchers can estimate the influence of different attributes on decision making.^14^

This study used a DCE to investigate how members of the Australian public trade off between different diagnostic testing options and service delivery factors when presented with symptoms related to oesophagogastric (OG), bowel, or lung cancer.

## Method

### DCE

Three separate DCEs were created for symptoms related to OG, bowel, and lung cancer, which are referred to as the OG cancer DCE, bowel cancer DCE, and lung cancer DCE. The methods for this study were based on recommended research practices for stated preference methods.^15^^–^^17^ Ethics approval was granted by the University of Melbourne Human Research Ethics Committee (HREC ref. no. 2022-25008-32501-4).

### Scenarios and attributes

For each DCE three scenarios were devised to portray distinct probabilities of having undiagnosed cancer, encompassing symptom positive predictive values (PPVs) of around 1% to 3%, referred to herein as low-risk, moderate-risk, and higher-risk scenarios (Table 1). These scenarios were based on risk-assessment tools generated from the UK-based CAncer Prediction in ExeteR (CAPER) studies.^18^^–^^20^ In the current study the authors focused on symptoms with lower PPVs to understand possible variations in patient preferences at lower cancer risk levels. After conducting a formative qualitative study^21^ and reviewing existing literature, five attributes were selected including the testing strategy, familiarity with the GP, waiting time to have the test and receive the results, travel time for the test, and the test cost (Box 1).

**Table 1.** Symptom scenarios and attributes: symptom scenarios are organised by cancer type along with their corresponding PPVs for low-, moderate-, and higher-risk symptoms

**Cancer type and scenario**	**Symptom PPV (95% CI)**
**Oesophagogastric cancer**	
For the past 6 weeks, you have had **indigestion that comes and goes**.^a^ After describing your symptoms, your GP gives you three options:	0.7 (0.6 to 0.7)^18^
For the past 6 weeks, you have had **indigestion** that comes and goes as well as **nausea and vomiting**. After describing your symptoms, your GP gives you three options:	1.3 (0.9 to 1.8)^18^
For the past 6 weeks, **you have had indigestion that comes and goes and have lost some weight without trying**. After describing your symptoms, your GP gives you three options:	2.1 (1.3 to 3.5)^18^

**Bowel cancer**	
For the past 6 weeks, you have had **diarrhoea** on most days. After describing your symptoms, your GP gives you three options:	0.9 (0.7 to 1.1)^19^
For the past 6 weeks, you have had **diarrhoea and stomach cramps** on most days. After describing your symptoms, your GP gives you three options:	1.9 (1.4 to 2.7)^19^
For the past 6 weeks, you have had **diarrhoea** on most days and have **lost some weight without trying**. After describing your symptoms, your GP gives you three options:	3.1 (1.8 to 5.5)^19^

**Lung cancer**	
For the past 6 weeks, you have been **coughing** most days and **feel more tired** than usual. After describing your symptoms, your GP gives you three options:	0.6 (0.5 to 0.9)^20^
For the past 6 weeks, you have been **coughing on most days** and have **lost some weight without trying**. After describing your symptoms, your GP gives you three options:	1.8 (1.1 to 2.9)^20^
For the past 6 weeks, you have **felt more tired than usual** and have **coughed up blood on one occasion**. After describing your symptoms, your GP gives you three options:	3.3^20^^,^^b^

a *Bold was used in the original survey to emphasise the changing symptoms.*

b *No CI available. CI = confidence interval. PPV = positive predictive value.*

**Box 1.** Symptom attributes^a^

**Attribute and survey description**	**Level**
**Testing strategy**	
The type of test (or initial treatment) is: *(OG cancer DCE)*	A medication that lowers your stomach acid (proton pump inhibitor, for example, Nexium^®^)^b^ A breathing test for bacteria that can cause stomach ulcers (*Helicobacter pylori* breath test) A procedure where a camera inspects your stomach (gastroscopy)
The type of test (or initial treatment) is: *(Bowel cancer DCE)*	A test that looks for blood in your poo (faecal occult blood test [iFOBT])^c^ A CT scan of your abdomen A procedure where a camera inspects your bowel (colonoscopy)
The type of test (or initial treatment) is: *(Lung cancer DCE)*	A course of antibiotics for 5 days A chest X-ray A CT scan of your lungs

**GP relationship**	
The GP you are seeing about this is:	A new GP at a practice you have not been to before A new GP at the usual practice you attend Your regular GP at the usual practice you attend

**Waiting time for testing and receiving the results**	
The waiting time for your test and results is:	Up to 2 weeks 2–8 weeks Over 8 weeks

**Travel time to testing location**	
The travel time to have your test is:	Up to 20 min 20–60 min Over 60 min

**The test cost**	
The out-of-pocket cost is:	$0 $75 $150

a *The ‘testing strategy’ attribute provides information about investigations for each cancer type that are referred to as the least invasive, more invasive, and most invasive tests. The remaining attribute levels remain consistent across all three cancer DCEs.*

b *Nexium^®^ is manufactured by AstraZeneca.*

c *The Australian iFOBT is equivalent to the UK faecal immunochemical test. $ = Australian dollars. CT = computed tomography. DCE = discrete-choice experiment. OG = oesophagogastric.*

### Experimental and survey design

A D-efficient fractional factorial design for two unlabelled alternatives and an opt-out option was produced using the experimental design software Ngene (ChoiceMetrics, 2021).^22^ The design was optimised to estimate a conditional logit model and examine the main effects and interactions between the scenarios and attributes. A 24-row design was divided into two blocks of 12 choice tasks to reduce participant burden. The design was constrained to present the base level of ‘test strategy’ only with the base levels of ‘waiting time’ and ‘travel time’ to ensure the options were realistic. The initial design was generated using assumed priors, which were limited to indicating the direction of effect (positive or negative) for each coefficient. This design was piloted with approximately 300 participants (100 participants per DCE) and the pilot data were then used to estimate priors for each DCE using a conditional logit model. Using the estimated priors, three final DCE designs were produced. The final experimental design of each cancer DCE differed because of the unique estimated priors generated from each pilot.

A ‘think-aloud’ pilot testing phase was conducted through video conference with two consumers affiliated with the Victorian Comprehensive Cancer Centre Alliance. During the testing, consumers completed the online survey while sharing their screens with a member of the research team (the first author). Any issues identified during the survey were discussed, documented, and addressed to improve clarity and functionality. The feedback provided by the consumers resulted in several changes to the survey, which are summarised in Supplementary Table S1.

The final DCE survey commenced with a screen asking participants to enter their age, location, and sex to confirm eligibility and fill quota sampling targets. This was followed by background information, a consent form, and an explanation of the attributes and levels. To help participants understand the process, there was an example and practice choice question. This was followed by 12 choice tasks, each displaying two testing options and an opt-out option. The opt-out involved no test and a GP review of the patient’s condition in 2–4 weeks if symptoms persisted.

**Figure 1.**
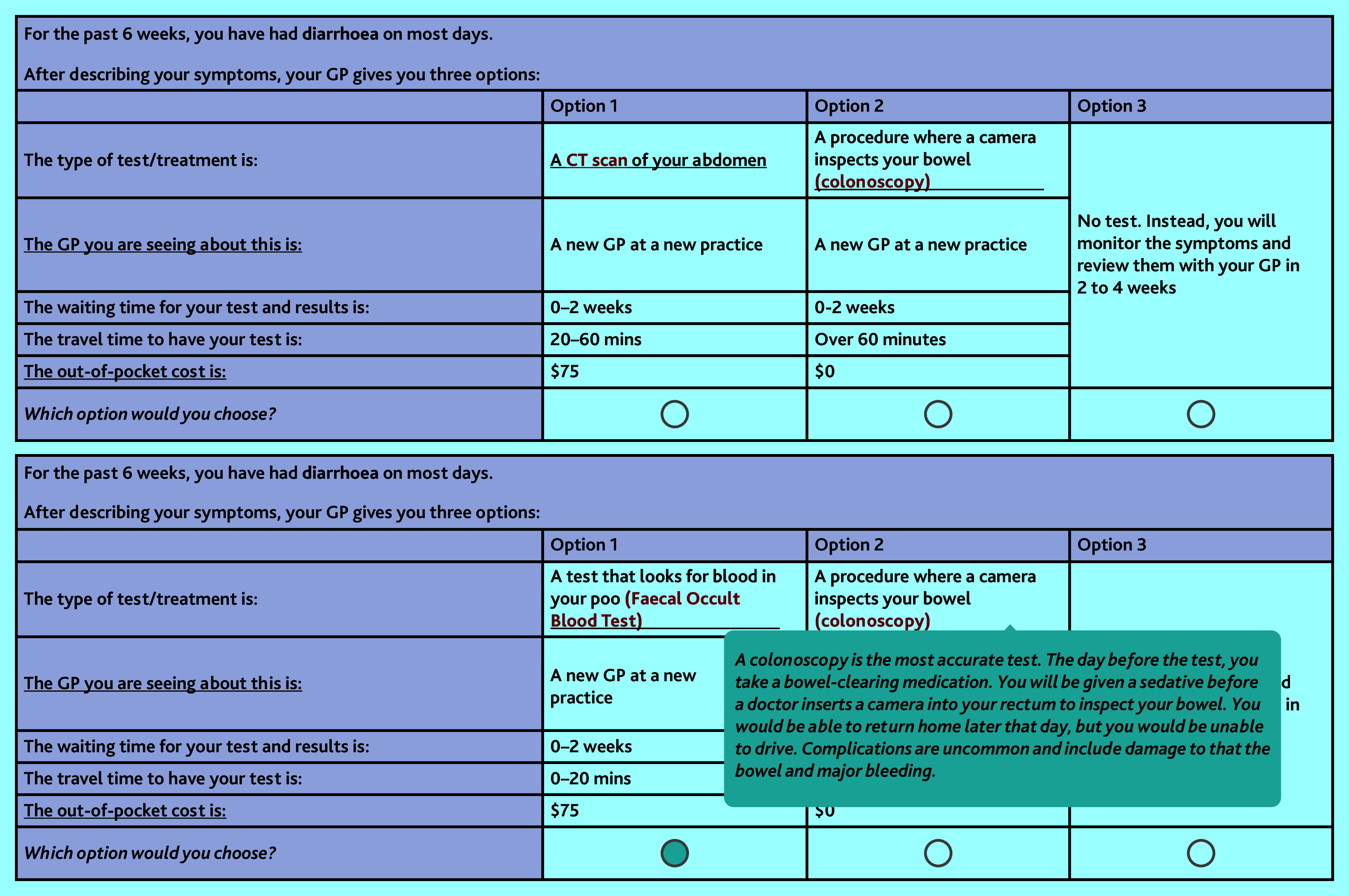
Sample question from the bowel cancer discrete-choice experiment and an example of a hover description for the colonoscopy. $ = Australian dollars. CT = computed tomography.

Across the 12 choice tasks each participant sequentially completed four questions featuring the low-risk symptoms, four questions presenting the moderate-risk symptoms, and four questions presenting the higher-risk symptoms. The scenarios described the symptoms but did not explicitly mention the symptom PPV. To enhance clarity, hover tools enabled responders to place their cursor over specific elements of the survey that would then provide additional details, such as the nature of investigations. The hover descriptions for each test or treatment are included in Supplementary Table S2. The survey concluded with questions about the difficulty of the DCE, reasons participants selected the opt-out option, and sociodemographic information such as participant employment status, income, and private health insurance status. Figure 1 illustrates an example choice question.

### Participants and recruitment

Participants were recruited through Pureprofile (http://www.pureprofile.com.au), an Australian online survey panel. Panel members registered with Pureprofile were invited to participate through the ‘feed’ on their account homepage. They were provided with information on the survey content, length, and payment. Participants were paid based on the time taken to complete the survey. Quota sampling was used to recruit a nationally representative sample of 3300 Australians (300 reserved for the pilot) aged ≥40 years. An age threshold of 40 years was selected based on the premise that cancer risk significantly increases beyond this point^23^ and to enable meaningful comparison with similar studies.^9^^,^^24^

### Statistical analysis

Data analyses were conducted using Stata 17 (StataCorp, College Station, TX, US). Descriptive statistics were used to summarise sociodemographic characteristics. Attributes were effects coded and then preferences were estimated using a series of conditional logit models.^17^ The first conditional logit model examined the main effects of the attributes. To determine whether the preference for opting out varied across scenarios, a second conditional model included an interaction term between the opt-out option and the three scenarios. To determine whether attribute preferences differed across scenarios, a third conditional logit model included interaction terms between each attribute level and scenarios two and three.

The relative attribute importance, that is, the comparative weight assigned to different attributes within the DCE, was determined by taking the range of coefficients for each attribute from the main-effects conditional logit model and dividing it by the sum of the ranges for all attributes within each experiment.^17^

The influence of sociodemographic variables on preferences was determined by interacting age, sex, rurality, income, education, private health insurance, and having a regular GP with each attribute in a series of main-effects conditional logit models.

A mixed logit (MXL) model was estimated for each DCE to examine preference heterogeneity. The main effects were included as random parameters, and the model was estimated using a maximum likelihood approach with 500 Halton draws. MXL models account for the panel nature of the data, relax the independence of irrelevant alternatives assumption, and allow the estimation of both the mean and standard deviation of effects across the sample.^17^ The statistical significance and magnitude of the standard deviation estimates for each random parameter provide insight into the degree of variability in preferences.

## Results

### Participants

A total of 3013 people completed one of three surveys: 1004 for the OG cancer DCE, 1006 for the bowel cancer DCE, and 1003 for the lung cancer DCE. Across the three surveys, the response rate (that is, how many people started the survey compared with how many were invited to do it) averaged 4758/9064 (52.5%), and the completion rate (that is, how many people finished the survey compared with how many started it, including those who were screened out or reached the quota) averaged 3013/4758 (63.3%). The sociodemographic characteristics of the three groups of participants are summarised in Table 2.

**Table 2.** Participant characteristics across the OG, bowel, and lung cancer DCE^a^

**Characteristics**	**OG cancer DCE (*n* = 1004)**	**Bowel cancer DCE (*n* = 1006)**	**Lung cancer DCE (*n* = 1003)**	**Australian population,^a^ %**
**Sex^b^**				
Male	490 (48.8)	498 (49.5)	493 (49.2)	49
Female	513 (51.1)	507 (50.4)	509 (50.7)	51
Other	1 (0.1)	1 (0.1)	1 (0.1)	

**Age bands^b^**				
40–49 years	308 (30.7)	315 (31.3)	307 (30.6)	27
50–59 years	258 (25.7)	257 (25.5)	257 (25.6)	26
60–69 years	226 (22.5)	227 (22.6)	228 (22.7)	22
≥70 years	212 (21.1)	207 (20.6)	211 (21.0)	25

**State^b^**				
New South Wales	294 (29.2)	310 (30.8)	313 (31.2)	32
Victoria	247 (24.6)	250 (24.9)	254 (25.3)	25
Queensland	235 (23.4)	202 (20.1)	210 (20.9)	20
South Australia	84 (8.4)	93 (9.2)	71 (7.1)	8
Western Australia	95 (9.5)	96 (9.5)	100 (10.0)	11
Tasmania	22 (2.2)	28 (2.8)	30 (3.0)	2
Australian Capital Territory	20 (2.0)	20 (2.0)	21 (2.1)	2
Northern territory	7 (0.7)	7 (0.7)	4 (0.4)	1

**Remoteness area^c^**				
Major city	699 (69.6)	695 (69.1)	699 (69.7)	72
Regional and remote	305 (30.4)	311 (30.9)	304 (30.3)	28

**Indigenous Australian^b^**				
Yes	8 (0.8)	18 (1.8)	20 (2.0)	1
No	996 (99.2)	988 (98.2)	983 (98.0)	99

**Country of birth^b^**				
Australia	725 (72.2)	762 (75.7)	729 (72.7)	64
Other	279 (27.8)	244 (24.3)	274 (27.3)	36

**Educational attainment**				
School only	323 (32.2)	317 (31.5)	309 (30.8)	37
Vocational qualification	288 (28.7)	297 (29.5)	286 (28.5)	30
University qualification	393 (39.1)	392 (39.0)	408 (40.7)	33

**Smokes^d^**				
Yes	154 (15.3)	152 (15.1)	149 (14.9)	10
No	850 (84.7)	854 (84.9)	854 (85.1)	90

**Private health insurance^e^**				
Yes	575 (57.3)	570 (56.7)	606 (60.4)	45
No	429 (42.7)	436 (43.3)	397 (39.6)	55
**Regular GP**				
Yes	832 (82.9)	840 (83.5)	837 (83.4)	N/A
No	172 (17.1)	166 (16.5)	166 (16.6)	

**Survey completion time (min), means (95% CI)**	25 (16–33)	18 (15–21)	22 (18–27)	N/A

*Data are n (%) unless otherwise indicated.*

a *2021 Australian Bureau of Statistics (ABS).^25^*

b *For ABS data: age adjusted for ≥40 years.*

c *For ABS data: adult population >18 years.*

d *Currently smokes daily.*

e *Australian Prudential Regulation Authority March 2023 quarterly statistics. CI = confidence interval. DCE = discrete-choice experiment. N/A = not applicable. OG = oesophagogastric.*

Across all three DCEs, approximately one-fifth (630/3013, 20.9%) of participants were aged ≥70 years of age, one-quarter (797/3013, 26.5%) were born outside of Australia, 39.6% (1193/3013) had a tertiary education, 15.1% (455/3013) smoked, and 83.3% (2509/3013) reported having a regular GP. The sample was comparable with the Australian population in terms of gender, state, remoteness area, country of birth, and educational attainment but had a greater representation of higher-income earners and those with private health insurance. Participants reported a good understanding of the choice questions (Supplementary Table S3).

### Preferences for diagnostic testing

The results of the conditional logit main-effects model are summarised in Supplementary Table S4. Participants preferred more invasive tests (except in the OG cancer DCE for which the *Helicobacter pylori* breath test negatively influenced choice). The most favoured tests were:

- gastroscopy in the OG cancer DCE;
- colonoscopy in the bowel cancer DCE; and
- computed tomography chest scan in the lung cancer DCE.

There was a preference for a person’s regular GP and practice compared with seeing an unfamiliar GP in a new practice. Increased waiting time, travel time, and test cost negatively influenced preferences. The negative coefficient for the opt-out option indicates that participants were less likely to choose the no-test option and preferred to be tested. Supplementary Table S5 illustrates why participants chose to opt in or out of the test. The most common reasons for opting to be tested included early detection (1475/3013, 49.0%) and peace of mind (795/3013, 26.4%). Reasons for opting out of testing included the unpleasantness of the test (420/3013, 13.9%), perceived low risk of cancer (358/3013, 11.9%), and inconvenience (367/3013, 12.2%).

**Figure 2.**
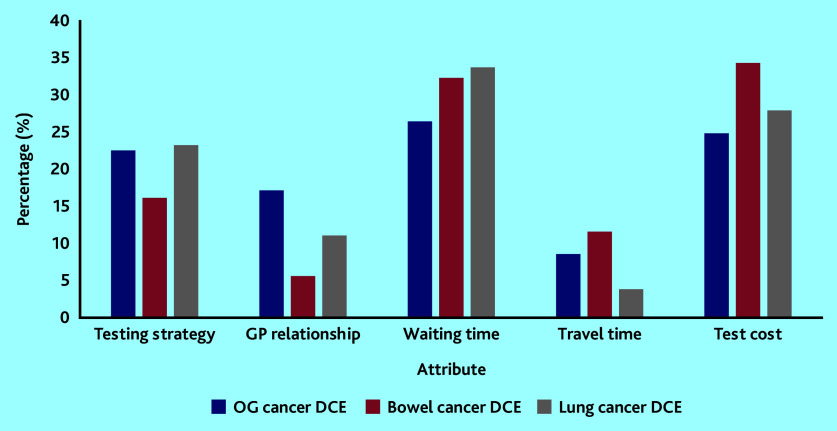
Relative attribute importance using coefficients derived from the main-effects conditional logit model. DCE = discrete-choice experiment. OG = oesophagogastric.

The relative importance of each attribute is illustrated in Figure 2. The attributes ‘waiting time’ and ‘test cost’ were the most influential followed by ‘test strategy’, whereas the least influential attribute was ‘travel time’. The ‘GP relationship’ attribute was more important in the OG cancer DCE than in the bowel and lung cancer DCEs.

Statistically significant interactions between DCE attributes and sociodemographic variables are displayed in Supplementary Table S6. Participants aged ≥60 years and those who reported having a regular GP preferred their regular GP. Participants aged >60 years and those with a tertiary education preferred more invasive forms of testing, such as a colonoscopy in the case of the bowel cancer DCE or gastroscopy for the OG DCE.

### The influence of the risk scenario on preferences

Interactions between the opt-out option and the low-risk, moderate-risk, and higher-risk scenarios suggest that participants were more inclined to choose the opt-out option when presented with the low-risk scenario (Table 3). On the other hand, participants were less likely to choose the opt-out option and hence favoured testing when presented with moderate- and higher-risk scenarios.

**Table 3.** Conditional logit model including interactions between the opt-out option and the symptom scenarios^a^

**Attribute and level**	**OG cancer DCE**		**Bowel cancer DCE**		**Lung cancer DCE**	

	**Coefficient**	**SE**	**Coefficient**	**SE**	**Coefficient**	**SE**
**Testing strategy**						
Least invasive	−0.15		−0.89		−1.52	
More invasive	−0.37	0.08	0.43^b^	0.06	0.39	0.07
Most invasive	0.52^b^	0.08	0.45^b^	0.07	1.13^b^	0.07

**GP relationship**						
New GP, new practice	−0.67		−0.30		−0.78	
New GP, regular practice	0.12^b^	0.40	−0.11^c^	0.05	0.25^b^	0.00
Regular GP, regular practice	0.55^b^	0.05	0.41^b^	0.05	0.52^b^	0.04

**Waiting time**						
Up to 2 weeks	1.05		1.81		2.47	
2–8 weeks	−0.37^b^	0.03	−0.45^b^	0.04	−0.87^b^	0.04
Over 8 weeks	−0.68^b^	0.04	−1.36^b^	0.04	−1.60^b^	0.06

**Travel time**						
Up to 20 min	0.58		0.74		0.27	
20–60 min	−0.22^b^	0.05	−0.29^b^	0.04	−0.19^b^	0.03
Over 60 min	−0.36^b^	0.04	−0.45^b^	0.04	−0.08	0.05

**Test cost**						
$0	3.06		2.18		2.12	
$75	−1.13^b^	0.05	−0.76^b^	0.05	−0.82^b^	0.05
$150	−1.93^b^	0.07	−1.42^b^	0.06	−1.30^b^	0.06

**Opt-out**	−0.68^b^	0.10	−1.21^b^	0.09	−0.93^b^	0.07

**Opt-out and scenario interactions**						
Low-risk scenario	1.10		1.27		1.44	
Moderate-risk scenario	−0.54^b^	0.06	−0.61^b^	0.05	−0.46^b^	0.05
Higher-risk scenario	−0.56^b^	0.06	−0.67^b^	0.06	−0.98^b^	0.08

a *The coefficient for the reference group is calculated as the negative sum of the other coefficients. Standard errors are provided for each non-reference coefficient because these coefficients are directly estimated from the data.*

b P*<0.01.*

c P*<0.05. $ = Australian dollars. DCE = discrete-choice experiment. OG = oesophagogastric. SE = standard error.*

Interactions between attribute levels and the moderate- and higher-risk scenarios reveal a preference for more invasive tests across all three DCEs, except for the *H. Pylori* test in the OG cancer DCE, which showed a negative coefficient in the higher-risk scenario (Table 4). Under the moderate-risk scenario, all three DCEs demonstrated a preference for attending a regular medical practice, whereas this was only present in the bowel cancer DCE under the higher-risk scenario. The analysis showed people were more willing to travel >60 min with more severe symptoms.

**Table 4.** Conditional logit model with interactions between the attribute levels and the moderate- and higher-risk scenarios^a^

**Attribute and level**	**OG cancer DCE**		**Bowel cancer DCE**		**Lung cancer DCE**	

	**Coefficient**	**SE**	**Coefficient**	**SE**	**Coefficient**	**SE**
**Testing strategy**						
Least invasive	−0.07		−0.01		−0.44	
More invasive	−0.27^b^	0.05	−0.02	0.05	0.11	0.02
Most invasive	0.34^b^	0.03	0.03^c^	0.04	0.33^a^	0.03

**GP relationship**						
New GP, new practice	−0.28		−0.19		−0.43	
New GP, regular practice	0.01	0.03	−0.09^b^	0.05	0.15^b^	0.01
Regular GP, regular practice	0.26^b^	0.04	0.28^b^	0.05	0.28^d^	0.05

**Waiting time**						
Up to 2 weeks	0.37		0.49		0.46	
2–8 weeks	−0.05	0.05	−0.02	0.07	0.08	0.05
Over 8 weeks	−0.32^b^	0.05	−0.47^b^	0.07	−0.53^b^	0.08

**Travel time**						
Up to 20 min	0.00		0.21		0.44	
20–60 min	0.05	0.05	−0.06	0.04	−0.16	0.08
Over 60 min	−0.05	0.05	−0.15^b^	0.03	−0.28^b^	0.09

**Test cost**						
$0	0.91		0.82		0.67	
$75	−0.22^b^	0.04	0.01	0.04	0.54^c^	0.05
$150	−0.69^b^	0.04	−0.82^b^	0.05	−1.21^b^	0.07

**Opt-out**	−1.05^b^	0.10	−1.47^b^	0.09	−1.80^b^	0.09

**Moderate-risk scenario interactions**						
Least invasive	−0.04		−0.10		−1.62	
More invasive	−0.06	0.05	0.38^d^	0.09	1.24^b^	0.02
Most invasive	0.02^c^	0.03	0.28^d^	0.06	0.38^c^	0.02
New GP, new practice	0.18		−0.15		−0.97	
New GP, regular practice	0.33^b^	0.06	0.09	0.07	0.34	0.08
Regular GP, regular practice	−0.15	0.09	0.06^d^	0.07	0.63^b^	0.04
Up to 2 weeks	0.04		0.20		0.39	
2–8 weeks	−0.12	0.07	0.06	0.08	−0.36^b^	0.06
Over 8 weeks	0.07	0.09	−0.26^c^	0.09	−0.03	0.03
Up to 20 min	0.11		0.36		−0.21	
20–60 min	0.11	0.08	−0.32	0.06	−0.15	0.12
Over 60 min	−0.22^d^	0.11	−0.04^c^	0.07	0.36^d^	0.09
$0	−0.08		−0.40		−0.18	
$75	−0.01	0.04	0.14	0.06	−0.04	0.03
$150	0.08	0.05	0.26	0.06	0.22	0.05

**Higher-risk scenario interactions**						
Least invasive	0.07		−0.37		−2.60	
More invasive	−0.30^c^	0.02	0.15^d^	0.06	0.85^b^	0.09
Most invasive	0.23^d^	0.09	0.21^b^	0.06	1.75^d^	0.07
New GP, new practice	−0.02		0.12		0.03	
New GP, regular practice	−0.06	0.10	−0.23	0.09	0.18	0.11
Regular GP, regular practice	0.04	0.04	0.11^d^	0.06	−0.21^d^	0.07
Up to 2 weeks	−0.09		0.00		0.39	
2–8 weeks	0.10	0.07	0.04^b^	0.09	−0.03^b^	0.07
Over 8 weeks	−0.01^d^	0.06	−0.04^b^	0.08	−0.36^c^	0.09
Up to 20 min	0.32		0.15		0.02	
20–60 min	−0.04	0.05	−0.02	0.06	−0.15	0.09
Over 60 min	0.36^b^	0.04	0.13^d^	0.06	0.13^b^	0.05
$0	−0.09		−0.44		−0.11	
$75	−0.01	0.04	0.14^d^	0.05	−0.01	0.05
$150	0.11	0.06	0.31^b^	0.07	0.12	0.05

a *The coefficient for the reference group is calculated as the negative sum of the other coefficients. Standard errors are provided for each non-reference coefficient because these coefficients are directly estimated from the data.*

b P*<0.01.*

c P*<0.05.*

d P*<0.1. $ = Australian dollars. DCE = discrete-choice experiment. OG = oesophagogastric. SE = standard error.*

### Preference heterogeneity

A limited number of parameters displayed heterogeneity within each cancer DCE (Supplementary Table S7). There was a statistically significant standard deviation for the test cost level of Australian dollars ‘$75’ in the OG cancer DCE. Significant standard deviations were observed in the bowel cancer DCE for the ‘most invasive test, ‘regular GP, usual practice’, and ‘opt-out’ option. The ‘more invasive test, ‘any GP and usual practice’, and the test cost of ‘$150’ exhibited significant standard deviations in the lung cancer DCE.

## Discussion

### Summary

To the authors’ knowledge, this is the first published DCE that explores preferences for diagnostic testing strategies concerning common cancer-related symptoms in primary care. Health system factors, waiting time, and test cost most influenced decision making across all three DCEs scenarios. Although the test strategy was less important, there was a preference for more invasive tests. Participants were more likely to opt out of testing when presented with the lowest-risk symptoms equivalent to a symptom PPV of approximately 1%. This highlights the tension between patient preferences, particularly when they do not align with clinical guidelines, and the need for timely and affordable testing amidst health system pressures.

### Strengths and limitations

By presenting symptom scenarios that embodied distinct cancer PPVs without explicitly disclosing them to participants, the authors of the current study were able to tease out a limitation in comparable studies that struggled to determine whether participant preferences were influenced by the symptom description or by the stated risk level.^9^^,^^24^ Another notable strength is the rigorous approach taken to develop the DCE. The attributes were derived from formative qualitative research, and pilot testing was conducted of the survey online and with consumers, to ensure the clarity and relevance of the study. Although the 12 choice questions presented different scenarios, most participants reported a good understanding of the survey, indicating an acceptable level of difficulty. The study sample was also sizable, diverse, and comparable with the general population across most parameters, providing good generalisability.

However, using an online survey panel introduced sampling bias by selecting only participants who were already online survey users. Also, as the authors did not have access to the sociodemographic data of those who were invited to participate in the survey but declined, it was not possible to characterise the influence of sociodemographic variables on non-response patterns. Although the response rate was strong, the survey completion rate of 63% may be considered a weakness of this study. Additionally, the survey was only provided in English, which excluded individuals with limited English proficiency.

In terms of the structure of the DCE, the ordinal presentation of scenarios may have suggested that the symptom risk was increasing and this may have affected participant preferences. Finally, the current study findings assert that patient willingness to undergo testing is influenced primarily by participant attitudes toward different symptomatic presentations. This assumption may not translate to the real world as individuals genuinely concerned about their symptoms can readily access information about associated cancer risks online or through guidelines. This informed subset of patients represents a significant minority that should not be disregarded.

### Comparison with existing literature

As indicated by the relative attribute importance, the current study underscored that access-related factors, specifically waiting times and testing costs, were the most significant elements influencing preferences. Although the literature on cancer screening highlights the influence of test attributes such as efficacy, process, and cost on decision making, the impact of service delivery attributes such as waiting time and travel have been less well studied and reveal mixed results.^7^^,^^8^ A DCE examining patient preferences for GP appointments across high- and low-risk cancer symptoms found waiting times were more important than the duration and convenience of the consultation.^24^ Although shorter waiting times are intuitively preferable, the level of importance participants placed on this in the current study highlights the potential conflict between growing demands for testing and the desire for shorter waiting times. This conflict assumes even greater significance as health systems strive to recover from the impact of the COVID-19 pandemic while simultaneously preparing for an anticipated surge in patients with cancer.^26^^,^^27^ Additionally, increased testing through expanded primary care access will add to demand as will any follow-up imaging of incidental findings.^28^ Increased access to testing must be accompanied by increases in both the diagnostic workforce and facilities to avoid diagnostic delays through ballooning waiting times.^28^

In the current study, higher costs negatively affected choice. In Australia, patients may face out-of-pocket expenses for tests such as endoscopy through private referrals, although this is often covered at least in part by private health insurance. In fact, out-of-pocket expenses make up about 17% of healthcare spending, with diagnostic imaging ranking as the fourth-largest contributor.^3^ This scenario is also relevant to the UK where patients are increasingly opting to pay privately for quicker access, even without insurance.^29^ Like in Australia, this trend will disproportionately affect disadvantaged segments of the population.^30^

Although the testing strategy was not the foremost determinant of patient choice, this study highlights a clear preference for what the authors of the current study have classified as more invasive tests, but the precise drivers of this preference remain unclear. One plausible explanation is that participants might prefer these more invasive tests because of a perceived heightened accuracy, which would be consistent with findings elsewhere.^11^ This underscores the intricate interplay between perceived cancer risk, the perception of test accuracy, and patient preferences.

A study by Banks *et al* (2014) reported that 90% of participants opted to be tested for symptoms with 1% PPVs; however, the specific risk of cancer was stated in each scenario.^9^ In the current study a more clinically grounded approach was embraced by presenting various symptom severities without specifying symptom PPVs. Two primary factors guided this decision. First, evidence points to the limited use of such information by clinicians.^31^ This is likely to be even lower in the absence of risk-assessment tools, as observed in settings like Australia. Second, although the current research revolves around common symptoms viewed through a cancer lens, it is crucial to recognise and address the inherent bias stemming from the authors’ perspective as cancer researchers. In the practical realm of clinical practice, where these symptoms are routine and the actual risk of cancer is minimal, the inclination to frequently consider or highlight cancer and specific PPVs in discussions with patients experiencing such symptoms is likely to be quite low. To mitigate this bias, the authors deliberately avoided explicitly using the term ‘cancer risk’ in association with common low-risk symptoms, which could potentially sway preferences toward testing at low-risk PPVs. Nonetheless, the current study design still enabled us to identify an approximate PPV threshold at which diagnostic safety netting may be acceptable to patients. Although the study found most patients opted not to be investigated at the 1% level, results from the scenarios support the notion that patients often want to be investigated at PPV thresholds lower than the current 3% threshold recommended in the UK’s National Institute for Health and Care Excellence guidelines for urgent investigation.^32^

The current finding that participants preferred opting out for the lowest-risk symptoms emphasises the importance of considering diagnostic safety netting as a tool for addressing low-risk symptoms. Diagnostic safety netting in general practice involves systematic follow-up of patients with planned investigations for persistent symptoms.^33^ Safety netting can occur as an initial diagnostic strategy for low-risk or vague symptoms or to monitor symptoms when initial test results are normal.^33^ However, effective safety netting must be active, requiring alignment between patients and clinicians as well as precise follow-up mechanisms to prevent diagnostic delays.^33^^–^^35^ Innovative methods to improve safety netting through text messaging^36^ and co-designed action plans^37^ have been investigated. However, system-based approaches that incorporate proactive monitoring, information technology, and involvement of the broader healthcare team are likely to prove more holistic and effective.^38^ Nevertheless, safety netting alone may not be deemed satisfactory for patients seeking reassurance for their symptoms. In such cases, the current study results suggest alternative first-line tests such as faecal immunochemical testing (FIT), which is not currently recommended for symptomatic patients in Australian primary care, might be acceptable. This is consistent with other recent findings regarding the investigation of low-risk lower GI symptoms.^39^

Although sociodemographic factors had some influence on preferences for diagnostic testing, their impact was not consistent across different cancer DCEs. In the current study, participants aged ≥60 years expressed a preference for GI endoscopies in both the OG and bowel cancer DCEs. However, the influence of age on testing preferences has yielded mixed findings in the literature. A recent review found that older patients seek help sooner when they notice signs they attribute to cancer;^40^ however, this does not necessarily translate to a preference for more intensive investigations. Although Banks *et al* in 2014 found individuals aged 60–69 years had a stronger preference to be investigated, it was lowest for those aged ≤70 years.^9^ Conversely, in a cross-sectional survey conducted by Delisle *et al* in 2022, patients aged <65 years were more inclined to choose colonoscopy over FIT testing compared with other age groups.^39^

In the current study the authors expected that the rurality of participants would influence their preferences regarding the travel time attribute; however, this was not the case. This contrasts with research on help-seeking behaviours, which has identified the burden of travel as a significant factor contributing to delayed care among rural populations.^41^ The current study may have been limited by the relatively low travel times (<20 min, 20–60 min, and >60 min) incorporated into the travel time attribute, as participants in rural areas in Australia may be accustomed to travelling further for their healthcare needs.

### Implications for research and practice

Finding the right balance between early cancer diagnosis and appropriate investigations is a challenge in health systems with flexible gatekeeping roles and limited capacity. Clinicians should be empowered to engage in shared decision making about testing options and safety-netting measures for low-risk symptoms. Access to investigations at low-risk thresholds will be accompanied by public expectations for timely testing, and system capacity must effectively meet increased demands. Future research should explore diagnostic safety-netting strategies and the use of triage tests in primary care, particularly for low-risk symptoms, and focus on capacity building to ensure timely and accessible diagnostic services.
